# Experience gained from the implementation of the Saudi TraumA Registry (STAR)

**DOI:** 10.1186/s12913-019-4881-8

**Published:** 2020-01-06

**Authors:** Jane E. FORD, Abdulrahman S. ALQAHTANI, Shatha A. A. ABUZINADA, Peter A. CAMERON, Mark C. FITZGERALD, Ahmed S. ALENIZI, Dina FARJOU

**Affiliations:** 1 Department Epidemiology and Preventive Medicine, Monash University Level 3, 553 St Kilda Road, MELBOURNE, 3004 Australia; 2 Vision Realisation Office, Ministry of Health, Al Malaz, RIYADH, 12641 2219 Saudi Arabia; 3 King Saud Medical City, Al Imam Turki Ibn Abdullah Ibn Muhammad, Ulaishah, RIYADH, 12746 Saudi Arabia; 4 Department Epidemiology and Preventive Medicine, Monash University, Level 3, 553 St Kilda Road, MELBOURNE, 3004 Australia; 5 National Trauma Research Institute, Burnett Building, 85-89 Commercial Road, MELBOURNE, 3004 Australia; 6 Monash University, Wellington Road, CLAYTON, 3800 Australia

**Keywords:** Registries, Epidemiological Monitoring, Trauma Severity Indices, Benchmarking, Data Collection

## Abstract

**Background:**

Trauma registries are essential to trauma systems, to enable collection of the data needed to enhance clinical knowledge and monitor system performance. The King Saud Medical City (KSMC) in Riyadh, Kingdom of Saudi Arabia (KSA) is aiming to become a Level 1 Trauma Centre, and required a trauma registry in order to do so. Our objective was to establish the Saudi TraumA Registry (STAR) at the (KSMC) and ready it for national deployment. The challenge was that no formal trauma data collection had occurred previously and clinicians had no prior experience of trauma registries.

**Methods:**

To develop the registry, a novel 12 step implementation plan was created and followed at the KSMC. Registry criteria and a Minimum Dataset were selected; training was delivered; database specifications were written; operating procedures were developed and regular reporting was initiated.

**Results:**

Data collection commenced on August 1st 2017. The registry was fully operational by April 2018, eight months ahead of schedule. During the first year of data collection an average of 216 records per month were entered into the database. An inaugural report was presented at the Saudi Trauma Conference in February 2019.

**Conclusions:**

The strategy deployed at the KSMC has successfully established the STAR. In the short term, process indicators will track the development of the hospital into a Level 1 Trauma Centre. In the medium to long term the STAR will be rolled out nationally to capture the impact of public health initiatives aimed at reducing injury in the KSA. The effect of the STAR will be that the country is better equipped to deliver continuous improvements in trauma systems and quality of care.

## Background

Injuries are a significant cause of death and disability worldwide, regardless of the development status of the country [1]. The World Health Organization (WHO) reported on the magnitude of the entire problem at the Global Forum on Trauma Care in 2009 [2], and also declared the targeted Decade of Action for Road Safety 2011–2020 [3]. Trauma systems have been developed to address this growing threat to public health, of which the Victorian State Trauma System (VSTS) is a world-class example. A key component system wide monitoring and governance, based on accurate data from the state trauma registry. Following implementation in 2000, risk adjusted mortality decreased by 50% [4].

The Kingdom of Saudi Arabia (KSA) is the largest Arab country in the Middle East and is highly developed. It has a rapidly growing population estimated at 32.4 million people in 2017 [5] but is suffering a well-documented rise in the incidence of serious injuries that occur on the roads, in the workplace and elsewhere [6, 7]. Health care is provided largely by the Saudi Arabian Ministry of Health (MOH) with 407 primary health care centres. Other health care sectors include university, military and private groups [8]. MOH hospitals provide emergency care at Emergency Departments and it is to these that most trauma patients are transported by a combination of Saudi Red Crescent Society ambulances, private ambulances, police or military vehicles and private transport.

In response to the increasing burden of traumatic injuries on the community, the MOH requested the King Saud Medical City (KSMC) to engage the Alfred Hospital and Monash University to assist with trauma service development, including a trauma registry program based on the Victorian model which could be scaled to the country as a whole. This study aimed to examine the process of establishing the registry at the hospital with a view to rolling the prototype out nationally. This was a challenge as the hospital had no previous involvement with a trauma registry and it required establishment without any prior experience from clinical staff.

KSMC is located in the capital city, Riyadh, which is home to approximately six million people. It is the MOH centre that receives the most emergency patients in the Kingdom [9] and is therefore the logical initial site for a trauma registry that ultimately will be rolled out nationally. Recent reports reveal that approximately 350 people a day present to the Emergency Department (ED) at the KSMC, a significant proportion of whom will be admitted to one of the facility’s 1400 beds. Over 300 people per month presented with the level of trauma that potentially fell within the population of interest for a registry. To capture these data accurately required considerable effort and a robust methodology.

## Methods

### Work plan

A plan was created by the project team, based on the operating principles in the 2008 Australian Commission on Safety and Quality in Healthcare (ACSQH) [10], that stipulated 12 steps (Table 1).

**Table 1:** Trauma registry implementation 12 step plan

Step	Rationale
Step 1. Select inclusion criteria.	Trauma registries must consider the population of interest and determine the characteristics of eligible cases. It is not practical for a registry to collect all cases, as the burden and the cost of data collection would very quickly outweigh the benefit of the information gained from trivial or minor injuries.
Step 2. Select exclusion criteria.	It is equally important to select appropriate exclusion criteria, as this will assist in identifying cases of interest and increase the likelihood that only cases that meet criteria are registered on the database. Similar to the inclusion criteria, they must be clearly documented and applied rigorously.
Step 3. Select Minimum Dataset (MDS).	Once the population of interest has been considered and the inclusion and exclusion criteria have been identified, the Registry must select data items that will best capture the required information. Data items are otherwise known as variables, data elements or data points. The items must then be documented in a data dictionary that unequivocally defines each element and provides the rules for use.
Step 4. Identify data sources.	Selected data items must be readily available from the medical record or existing hospital systems. To ensure consistent data collection practices and reproducible data, the source of each item must be identified so that the same piece of information can be obtained from the same place for every case. The source should be documented in operational documents for ongoing reference by Registry staff.
Step 5. Determine method of collection.	Data collection methods depend on the existing systems at the facility; the processes that are already in place and whether it is undertaken in real time during the patient admission or retrospectively after discharge. The use of a paper form is the simplest method of data collection and arguably the most practical for a newly established registry. The hard copy form is usually completed by the data collector according to an agreed process, and then subsequently entered into a database at a later time. The advantage of this method is that paper is portable; data entry can be undertaken at any place and any time and does not need sophisticated technical input. Disadvantages include that it is difficult to store appropriately and the information is more vulnerable to human error due to the longer journey from source to database.
Step 6. Determine data collection personnel.	The personnel who collect data must understand the importance of the task, and prioritise it accordingly. Clinical staff will already have some training and understanding of the information they are required to collect, but may not prioritise the task when they have a competing priority to deliver clinical care to the patient. Non-clinical staff may be able to dedicate themselves to the task more completely, but they will require a greater degree of training to enable them to understand the data properly and perform effectively. The decision on who should collect the data is multifactorial and may depend on the outcome of Steps 4 and 5.
Step 7. Decide on reporting requirements.	The Registry will require a suite of reports that will meet its operational and research needs. These reports will enable monitoring of all aspects of trauma service activity at the facility, as well as provide information that drives research. The reporting functionality of a database should be flexible and be able to be generated routinely as well as on an as needs basis.
Step 8. Determine database functionality.	Registries require a system that will accept, store and report the data securely. They have a choice whether to acquire a product externally or build a product internally. If the system is built internally it requires a Systems Requirement Specification (SRS) that documents the business needs of the registry and the functionality required to meet those needs.
Step 9. Construct the database.	If the facility chooses to build a system internally, the primary reference for the software developers will be the data dictionary, previously referred to in Step 3. The system will be configured to the functionality previously determined in Step 8.
Step 10. Create a training program.	When the components of the registry are in place, the pre-determined data collection personnel must understand the purpose of the registry; be committed to the task of data collection and be trained to enter the data accurately and efficiently. A training program should be adjusted to suit the skill level of the participants.
Step 11. Create training materials.	Appropriate training materials should be available to data collectors, not only for use during the training program, but to be retained as a ready reference at all times. Training and reference materials include but are not limited to a data collection manual that provides clear instructions as to when, where and how the information is to be entered onto either the paper form or into the database; medical dictionaries, anatomical charts and other information appropriate to the skill level of the trainees.
Step 12. Deliver the training to data collectors.	The primary aim of a training program is to ensure that consistent data collection practices are in place and all personnel are cognisant of the agreed process. The format of the training should be appropriate to the environment at the facility and the learning style of the participants. Options for delivery of the training may be in a classroom format to a large group; a focus group format to a small group, or one on one. Once the training has been completed satisfactorily, and management is confident that data collection can commence, the implementation of the registry can be considered to be complete and ongoing operations can begin.

The initial steps were taken in sequence, with the latter steps taken either contemporaneously or out of sequence, depending on the progress at that time. Each step included a rationale and examples taken from other registries administered by the Alfred and Monash University, particularly the Australian Trauma Registry (ATR), the Victorian State Trauma Registry (VSTR) and the Alfred Health Trauma Registry (AHTR).

### Selection of the inclusion and exclusion criteria and Minimum Dataset (MDS)

Initial steps required the selection of inclusion and exclusion criteria and the selection of a Minimum Dataset (MDS). These are critical as the former identifies the target population of interest [11] and the latter specifies the required information, arguably forming the framework of the entire registry.

Our criteria were based on those of the VSTR and designed to include cases that added the most value to the knowledge gained from the registry, and exclude cases that the experience of the VSTR showed added minimal value (Table 2).

**Table 2:** STAR inclusion and exclusion criteria

Inclusion criteria	Exclusion criteria
1. Traumatic injury as reason for acute care and one or more of the following: • Death in the Emergency Department as a consequence of injury. • Inpatient death following injury. • Admission to the Intensive Care Unit.	1. Traumatic injury NOT the reason for acute care. 2. Any injuries distal to the wrist or ankle, except for amputation of the hand or foot at or proximal to the level of the metacarpals/metatarsals. 3. Length of stay < 3 calendar days apart from death and/or ICU admission. 4. Date of injury > 1 week prior to admission to the first hospital.

The literature reveals that other trauma registries have selected their MDS using a Delphi technique [12, 13], however our project chose to work with the trauma MDS collated by the Global Alliance for Care of the Injured (GACI). This option was available via the Monash University Accident Research Centre (MUARC), which is a World Health Organisation (WHO) collaborating centre. The MDS was in draft form at that time and has been referenced as such in the resultant KSMC data dictionary.

The final dataset necessarily included some variables that were specific to the KSA, evolving from an initial selection of 67 elements to the final listing of 83 elements. A significant number of these are drawn from those proposed by the WHO GACI working group. Additional File 1 lists each variable, the dictionary reference and dictionary definition.

### Site visits

Five site visits by the Alfred/Monash project team were necessary to implement the registry. Both visits included activities that were targeted at taking steps on the plan. The first visit in April 2017 achieved steps one to six. The second visit in August 2017 delivered a two-day training course to the on-site team as per step 12. A training program and materials had been created during the intervening months to complete steps 10 and 11. The course curriculum included presentations on data, registries and injury coding that were considered sufficient for the basic knowledge needed to implement the registry.

At the time of the second visit, all the steps of the 12-point plan had been taken except for step nine, construction of the database. However, this did not preclude the commencement of data collection on paper forms, which began on Wednesday 16th August.

The third, fourth and fifth visits incrementally progressed the implementation of the registry, culminating with User Acceptance Testing (UAT) of the database in March 2018 and the official Saudi TraumA Registry (STAR) launch in April 2018.

### Construction of the database

There are guides available to organisations that provide details on technical expectations and standards of clinical registries [14]. However, the decision on which data collection system to adopt is subjective, and depends on the organisation’s needs and resources. The KSMC project team found that the primary differential in the choice of database was whether it was acquired externally, or was a bespoke system that was built internally.

Specifications for the database were prepared by the project team and provided to the KSMC IT Department. No programming code was provided, as this is particular to development of the software and would be unique to the construction.

Once the project team considered the arguments for and against building the database internally, and the IT Department assessed whether they had the technical expertise to do so, the decision was made to proceed. Construction commenced in August 2017.

### Timeline

**Fig. 1:**
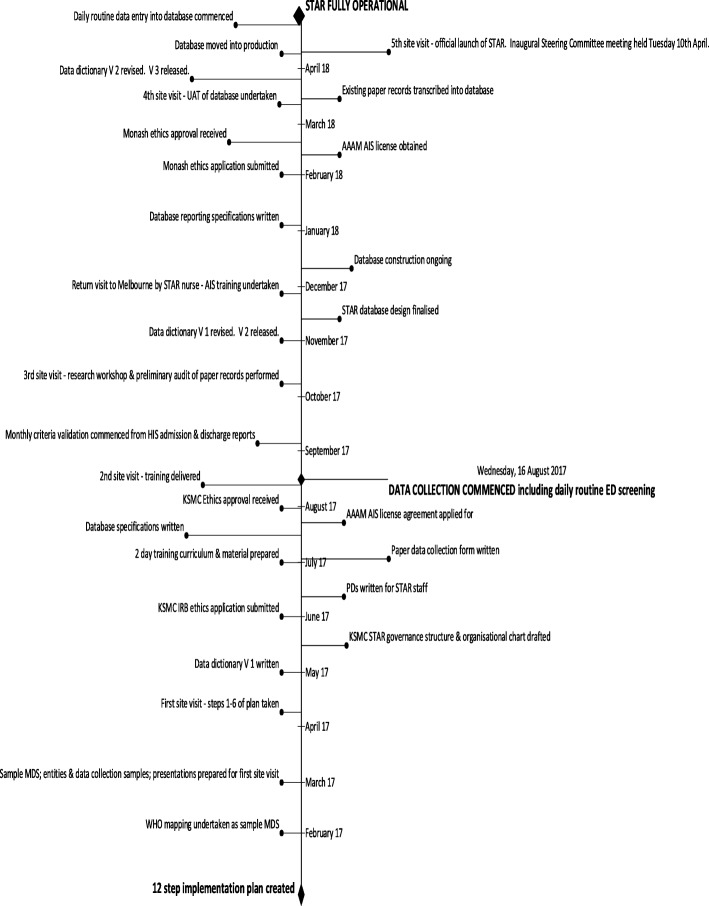
STAR implementation timeline

The time taken to implement the KSMC STAR was 16 months, from the commencement of project activities in January 2017 to the move into production of the database in April 2018 (Fig. 1).

### STAR operations

The day to day operations of registries differs between organisations according to the registry’s need and purpose. There are valuable recommendations in the literature [15] on the various strategies and methodologies, but as with the choice of data collection system there is no ‘one size fits all’. In the absence of any generic Standard Operating Procedures (SOPs) that would suffice, the STAR has written those necessary at this first site, that will also be applicable at ensuing sites.

An SOP was created for the methodology based on the experience of AHTR and the VSTR, which is that of retrospective data collection of cases that present to hospital and are found to meet inclusion criteria. The daily routine commenced on the 16th August. Cases were backdated to those with dates of injury beginning on the 1st August 2017 and the dataset was documented on paper forms initially. The database was ready for direct data entry in April 2018, after which time the registry was considered to be fully operational.

## Results

During the first year of data collection an average of 216 records per month were entered into the STAR database, screened from the approximately 300 cases of potential interest that presented to the KSMC ED. From this monthly average, 175 were confirmed to meet inclusion criteria and progressed to completion of the case. A data quality and cleaning regime was initiated within one month of reporting being available from the database, to immediately ensure prompt monitoring of data integrity. Inbuilt database edits disallowed blank fields, therefore no null values were found. The cleaning regime assessed completeness and quality as the proportion of ‘unknown’ values; and anomalous, or mismatched, values. Each report interrogated the injury event and definitive care variables, and has consistently revealed the variables of concern to be those relevant to the injury event; injury coding and operations coding (Table 3).

**Table 3:** Error rates of concerning STAR variables at one month post go live of the database

	n	% unknown	% anomalous/mismatched	% total errors
MRN	1803	0.00	0.00	0.00
Full name	1803	4.83	0.00	4.83
DOB	1803	0.00	0.50	0.50
Age at event	1803	0.00	0.22	0.22
Gender	1803	0.00	0.00	0.00
Injury date & time	1803	0.00	1.39	1.39
Event description	1803	0.00	10.43	10.43
Cause of injury	1803	0.67	14.03	14.70
Description if Cause is Other	9	0.00	77.78	77.78
Injury type	1803	0.06	1.55	1.61
Description if type is Other	2	0.00	0.00	0.00
Injury intent	1803	0.44	1.11	1.55
Place of injury	1803	25.51	0.22	25.73
Description if Place is Other	9	55.56	11.11	66.67
Activity at time of injury	1803	49.14	0.00	49.14
Activity description if Other	7	71.43	14.29	85.72
? Traffic event (activates counterpart fiels)	1803	0.00	10.65	10.65
Traffic counterpart	726	7.71	0.14	7.85
Geographical location of injury	1803	5.05	8.93	13.98
AIS codes	3479	N/A	7.70	7.70
Operation codes	891	N/A	66.78	66.78

There was a marked difference between the demographics variables, and the variables of concern, which had higher error rates. The sample size of ‘Descriptions if Cause/Place/Activity is other’ variables was small, however the rate reflects the consistent error of coding the value as ‘Other’, when in fact the value could have been captured in the codeset of the primary variable.

It is not possible to determine at which point newly implemented registries should be achieving a particular standard of data quality and completeness. What is agreed is that data quality should be optimised and that missing data is a known problem [16, 17]. These quality reports have provided insight into the work still needing to be done at any given time.

In addition to ongoing quality reports, a comprehensive audit in October 2018 interrogated approximately 10% of records from the first 12 months of operations. The resulting recommendations addressed the data quality issues and will ensure continuous improvement as the STAR matures.

An inaugural epidemiological report of the first 12 months of data collection has been prepared and was presented at the Saudi Trauma Conference held in Riyadh in February 2019. Ongoing annual reports of calendar years are planned.

## Discussion

The challenge facing the project team tasked with implementing a trauma registry at the KSMC was that trauma data had not formally been collected at the site previously, and there was no existing local example of how to do so. Despite this, the STAR has been successfully established at the KSMC and was fully operational eight months ahead of the project deliverable date of December 31st 2018. The 12 step implementation plan that was especially created for the project provided the framework for the process. Each step gave the project team confidence to proceed to the next step or schedule the required tasks accordingly. This process may be useful for other jurisdictions.

Through its partnership with the Monash University Collaborating Centre, the STAR was able to refer to the WHO draft trauma MDS for assistance in selecting its variables. KSMC has made a contribution to the WHO goal of promoting “wider and more standardised collection of data for injury surveillance and trauma care quality improvement.” [18] The education curriculum that was developed to meet the initial training needs of the STAR personnel; the specifications for the construction of the database and the myriad of other tasks that were required have showcased our plan as a project that the WHO can consider to be an example of a case study of good implementation practice. Collaboration will be ongoing as the project continues.

The timeline of the implementation showed the constant tasks required to implement the registry. Some were done by the Alfred-Monash team; some were done by the KSMC; some were done on site in Riyadh and some of them were done off site in Melbourne. The successful completion of each task contributed to the completion of the next and no single task could be achieved in isolation from another.

The project team were required to make a difficult decision on which data collection system would meet the KSMC’s needs. Choosing the option to construct the database on site has proven to be the correct one, as the system is purpose built and amenable to an upgrade that will enable it to accept data from multiple sites, thus meeting the needs of a national registry.

Of local significance is that for the first time the hospital has access to data that will inform process indicators such as time spent in the Emergency Department; rate of trauma team activation; time to operation and length of stay in the Intensive Care Unit. This information alone will assist the KSMC with its goal of achieving verification as a Level 1 Trauma Centre.

Of national significance to the KSA is that the STAR will mature to the point where it can be rolled out across the country, offering robust operating procedures and a functional database. The multi-site experience of the VSTR and the ATR will support the KSMC to be the locus for registry activity elsewhere in the greater Riyadh region and across the Kingdom. Last but not least, neighbouring countries will benefit from the knowledge gained of the implementation process from KSMC.

The literature [19] identifies several features of successful registries including but not limited to organisational support; existence of a steering committee; viable funding options; surety of purpose; committed registry management; training of registry staff; attention to data quality; organised data collection; secure data storage system and flexible reporting options. However, to the best of our knowledge the literature has not described any other step-by-step guide used in the establishment of other trauma registries and indeed O’Reilly et al [20] cited a perception among their study participants that no such guide was available. Our process may therefore be a first, and useful for other jurisdictions.

The STAR registry will enable robust assessment of outcomes and international benchmarking as it accrues increasingly high quality, accurate and accessible data that are available for interrogation and research.

## Conclusion

By taking a systematic approach to registry development, the KSMC- The Alfred International trauma Program has implemented a scalable regional trauma registry with robust and rigorous data collection, collation and potential to drive trauma system improvements. The STAR experience demonstrates essential development steps for implementation which will be effective in other regions.

## Supplementary information

**Additional file 1.** STAR Minimum Dataset. Full listing of dictionary data domain; dictionary reference; variable name and dictionary definition

## Data Availability

All data generated or analysed during this study are included in this article and its supplementary information file.

## Abbreviations

ACSQH: Australian commission on safety and quality in healthcare
AHTR: Alfred health trauma registry
ATR: Australian trauma registry
ED: Emergency department
GACI: Global alliance for care of the injured
IT: Information technology
KSA: Kingdom of Saudi Arabia
KSMC: King saud medical city
MDS: Minimum dataset
MOH: Ministry of health
MUARC: Monash university accident research centre
SOP: Standard operating procedure
STAR: Saudi trauma registry
UAT: User acceptance testing
VSTR: Victorian state trauma registry
VSTS: Victorian state trauma system
WHO: World Health Organisation
